# Repeated Cold Water Stress Leads to Improvements in Mitochondrial Metabolism of Skeletal Muscles in Rats

**DOI:** 10.3390/metabo16030179

**Published:** 2026-03-08

**Authors:** Mateusz Bosiacki, Maciej Tarnowski, Mariusz Panczyk, Anna Lubkowska

**Affiliations:** 1 Department of Biochemistry and Medical Chemistry, Pomeranian Medical University in Szczecin, Powstańców Wlkp. 72, 70-111 Szczecin, Poland; 2 Department of Physiology in Health Sciences, Pomeranian Medical University in Szczecin, Żołnierska Str. 54, 71-210 Szczecin, Poland; maciej.tarnowski@pum.edu.pl; 3 Department of Education and Research in Health Sciences, Faculty of Health Sciences, Medical University of Warsaw, 00-635 Warsaw, Poland; mariusz.panczyk@wum.edu.pl; 4 Department of Functional Diagnostics and Physical Medicine, Faculty of Health Sciences, Pomeranian Medical University in Szczecin, Żołnierska Str. 54, 71-210 Szczecin, Poland; anna.lubkowska@pum.edu.pl

**Keywords:** cold-water exercise, mitochondrial metabolism, lipid peroxidation, antioxidant enzymes, malate–aspartate shuttle (MAS), phosphofructokinase I (PFK-1), skeletal muscle

## Abstract

Background: In this study, we aimed to determine whether cold-water swimming could serve as a potential strategy to enhance antioxidant capacity, improve NADH utilization in oxidative metabolism, and consequently lead to better muscle metabolism and improved mitochondrial function in the skeletal muscles of rats. We hypothesized that cold-water swimming may upregulate malate–aspartate shuttle (MAS) expression, leading to more efficient NADH utilization in oxidative pathways and thereby improving muscle metabolism and mitochondrial function. Methods: We analyzed the expression of all MAS components, as well as the expression of phosphofructokinase I (PFK-1)—a key regulatory enzyme of glycolysis (which, under oxidative conditions, serves as a source of NADH for MAS)—in the skeletal muscles of rats subjected to cold-water swimming training. The study involved 32 male and 32 female rats aged 15 months, randomly assigned to control sedentary animals, animals training in cold water at 5 ± 2 °C, or animals training in water at thermal comfort temperature (36 ± 2 °C). The rats underwent swimming training for nine weeks, gradually increasing the duration of the sessions from 2 min to 4 min per day, five days a week. Results: Our findings revealed increased expression of all MAS enzymes involved in the delivery of NADH to mitochondria, elevated expression of the active form of PFK-1 indicating intensified glycolysis, increased reactive oxygen species (ROS) production, and upregulation of antioxidant enzymes. Conclusions: Cold-water swimming can improve metabolism and enhance mitochondrial function in the muscles of older adult rats subjected to cold-water swimming training.

## 1. Introduction

Regular physical activity is recommended to improve physiological and functional capacity in older adults. It has also been suggested that exercise training is associated with a 30% reduction in the risk of all-cause and cardiovascular (CV) mortality in individuals with and without preexisting CV disease [1,2,3]. In recent years, beyond the well-documented benefits of physical activity, growing attention has been directed toward the potential health-promoting effects of cold-water immersion—not only as a pre- or post-exercise cooling method but also in the form of winter swimming, which is increasingly practiced by the general public. A recent meta-analysis by Cain et al. (2025), a comprehensive assessment of the effects of cold-water immersion on the health and well-being of healthy adults, revealed a number of potential benefits, including reduced stress and improved sleep and quality of life [4]. However, according to the authors, these benefits remain unproven, with equivocal evidence regarding effects on immunity and mood and concerning findings regarding short-term increases in inflammation. The authors also emphasize that the current evidence base is limited by a small number of randomized controlled trials, small sample sizes, and a lack of diversity in study populations [4]. At the same time, people today frequently train in cold environments (e.g., low temperatures, strong winds, or cold-water immersion) in search of factors that enhance immunity and promote healthy aging through adaptive changes [5]. Furthermore, repeated exposure to cold water is thought to induce distinct acclimation patterns depending on temperature, exposure time, and length of the acclimation period [6]. These inconclusive results, combined with the growing public interest in cold-water swimming, highlight the need for caution and further research, including long-term studies and monitoring for adverse effects [4].

Mitochondrial metabolism and antioxidant defence mechanisms are generally considered to decline with age. One proposed protective mechanism in aging rats involves exercise-induced activation of antioxidant enzymes, particularly in skeletal muscle, as an adaptive response to increased formation of reactive oxygen species (ROS) that accompanies elevated oxygen consumption and improved mitochondrial energy metabolism during physical activity—especially in cold environments [7]. The impact of acute and chronic exercise on oxidative stress and antioxidant systems in aged organisms has been investigated in several studies in animal models [8] and humans [9,10,11]. Some evidence also suggests that even short-term cold-water immersion may provoke oxidative stress and elevate ROS levels, thereby triggering metabolic adaptations that enhance mitochondrial dynamics and ATP production during repeated exposures in animal models [7,12,13] and humans [14]. However, knowledge in this area—particularly at the cellular and tissue levels—remains incomplete.

The generation of free radicals increases with rising oxygen consumption and oxidative phosphorylation and is directly correlated with exercise intensity. While only a few studies have confirmed that cold-water swimming induces oxidative stress in humans [15,16,17], repeated cold-water immersion may help strengthen antioxidant defences during aging [18,19,20].

To test this hypothesis, we conducted a study evaluating selected markers of pro-oxidant/antioxidant status in response to cold-water exercise, compared to swimming under thermoneutral conditions and sedentary controls, in older adults male and female rats. We analyzed changes in skeletal muscle by measuring levels of two well-established in vivo lipid peroxidation markers, 8-isoprostane and malondialdehyde (MDA), as assessed by thiobarbituric acid reactive substances (TBARS) [19]. To investigate potential adaptive changes in antioxidant capacity, we measured the activity of key enzymes in the antioxidant system: superoxide dismutase (SOD1 or CuZn-SOD; E.C.1.15.1.1), catalase (CAT; E.C.1.11.1.6), glutathione peroxidase (GPx; E.C.1.11.1.9), glutathione reductase (GSSG-R; E.C.1.8.1.7), and glutathione S-transferase (GST; E.C.2.5.1.18).

**Figure 1:**
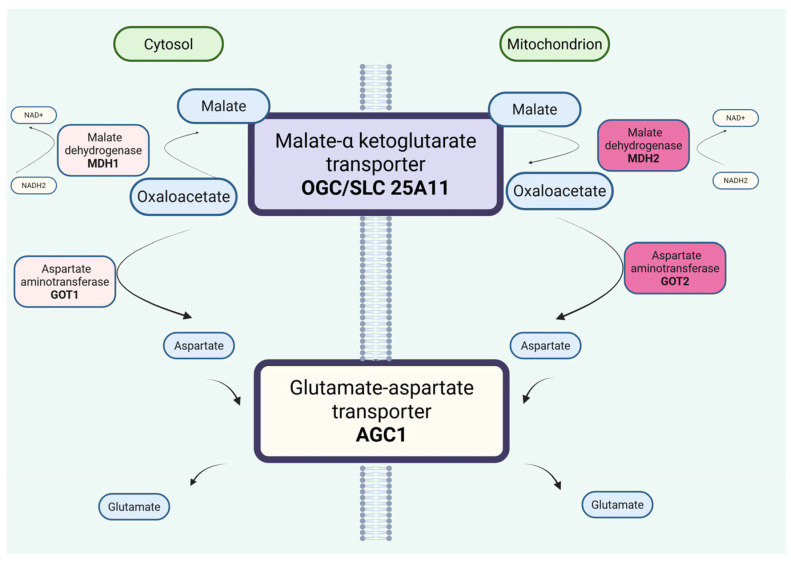
The malate–aspartate shuttle (MAS) plays a key role in maintaining redox balance in both mitochondria and the cytosol. The transport of reducing equivalents in the form of NADH across the inner mitochondrial membrane involves four enzymes and two mitochondrial carriers. MAS enzymes include cytosolic and mitochondrial NAD(H)-dependent malate dehydrogenases (EC 1.1.1.37; encoded by *MDH1* and *MDH2*) and cytosolic and mitochondrial aspartate aminotransferases (EC 2.6.1.1; encoded by *GOT1* and *GOT2*). The mitochondrial carriers include the alpha-ketoglutarate/malate carrier (OGC; gene *SLC25A11*) and two isoforms of the aspartate–glutamate carrier: *AGC1* (also known as Aralar; gene *SLC25A12*) and *AGC2* (also known as Citrin; gene *SLC25A13*) [23].

The malate–aspartate shuttle (MAS) plays a key role in maintaining redox balance in both mitochondria and the cytosol. Nutrients entering the cytosol are oxidized through various metabolic pathways by enzymes that use NAD^+^ as a cofactor. These pathways generate cytosolic reducing equivalents in the form of NADH, which must be transported into the mitochondria to support energy production. As the inner mitochondrial membrane is impermeable to NADH, MAS transfers cytosolic reducing equivalents across the membrane [21]. Ultimately, electrons are delivered to the electron transport chain in the form of NADH to support ATP synthesis, while MAS concurrently regenerates cytosolic NAD^+^. This cytosolic NADH reoxidation system maintains a compartmentalized NAD^+^/NADH balance, which is essential for cellular energy metabolism and drives NAD^+^-dependent cytosolic reactions such as glycolysis [22]. Hence, MAS functions as a redox transporter supporting oxidative metabolism and oxidative phosphorylation [23]. The transport of reducing equivalents across the inner mitochondrial membrane involves four enzymes and two mitochondrial carriers. MAS enzymes include cytosolic and mitochondrial NAD(H)-dependent malate dehydrogenases (EC 1.1.1.37; encoded by *MDH1* and *MDH2*) and cytosolic and mitochondrial aspartate aminotransferases (EC 2.6.1.1; encoded by *GOT1* and *GOT2*). The mitochondrial carriers include the alpha-ketoglutarate/malate carrier (OGC; gene *SLC25A11*) and two isoforms of the aspartate–glutamate carrier, *AGC1* (also known as Aralar; gene *SLC25A12*) and *AGC2* (also known as Citrin; gene *SLC25A13*), as seen in Figure 1 [23].

To date, no studies have investigated the effect of repeated cold-water immersion on MAS enzymes. We assume that cold-water swimming may upregulate MAS expression in older adult rats, leading to more efficient NADH utilization in oxidative pathways and thereby improving muscle metabolism, mitochondrial function, and ATP production—effects we previously observed [7]. In the present study, we also analyzed the expression of all MAS components, as well as the expression of phosphofructokinase I—a key regulatory enzyme of glycolysis (which, under oxidative conditions, serves as a source of NADH for MAS)—in the skeletal muscles of rats subjected to cold-water swimming training.

## 2. Materials and Methods

### 2.1. Animals

The experimental protocol received approval from the Local Ethical Committee on Animal Experimentation in Szczecin, Poland (Decision No. 38/2015 from 13 July 2015; Annex Bioethics Committee at the Pomeranian Medical University, Decision No. 008.236.2025, 17 December 2025), in full compliance with Directive 2010/63/EU for the protection of animals used for scientific purposes. All animal handling procedures adhered strictly to established international guidelines governing the care and use of laboratory animals, with deliberate measures implemented to limit animal numbers and alleviate distress where feasible. Efforts were made to minimise the number of animals used and to reduce potential suffering. The experiments were carried out on Wistar rats (Imp: EPI F) sourced from the breeding facility at the Prof. J. Nofer Institute of Occupational Medicine in Łódź, Poland, Laboratory for Research on Medicinal and Veterinary Products in the GMP Quality System (Łódź, Poland), which operates breeding of small rodents with the SPF standard. Animals were housed under controlled environmental conditions (12:12 h light/dark cycle, stable temperature) in enriched polycarbonate cages containing shelters, wooden chew blocks, bedding shavings, and nesting cotton. Daily health monitoring ensured early detection of any welfare compromise, while all interventions were scheduled between 08:00 and 12:00 h in a dedicated procedure room to standardize conditions and minimize stress. A detailed description of the experimental protocol has been published previously [7]. Briefly, sixty-four 15-month-old male and female albino Wistar rats were randomly assigned to three groups:

The first group consisted of sedentary aged controls: male (*n* = 8) and female (*n* = 8) rats.
The second group included aged rats trained by swimming in cold water (5 ± 2 °C): male (*n* = 12), female (*n* = 12).
The third group comprised rats swimming in thermoneutral water (36 ± 2 °C): male (*n* = 12), female (*n* = 12).

All animals were housed in groups of two to four per cage at 23 ± 2 °C, with 40% relative humidity and a 12:12 h light/dark cycle. They were fed standard rat chow and had access to tap water ad libitum.

#### Exercise Procedures

The duration of a single swimming session was selected based on literature data and our preliminary study, which show that at a water temperature of 4–5 °C rats actively swim for a maximum of 4 min, after which they begin to passively drift [24].

When swimming at a comfortable temperature, rats are active for significantly longer. Considering this, it was decided to use 4-min daily sessions for each experimental group, ensuring that the only factor differentiating the effects was the temperature of the water environment in which the animals swam. This model we also used in our previous studies [7,12,16].

The two exercise-trained groups (5 ± 2 °C and 36 ± 2 °C) swam for 4 min per day, five days per week, over eight weeks (between 9:00 and 11:00 a.m. on each training day). In the first week, the duration of each swimming session began at 2 min and increased by 0.5 min daily until reaching 4 min. The sedentary control rats were housed under identical conditions and handled as frequently as the experimental groups.

Swimming was conducted in glass tanks (100 cm long × 50 cm wide × 50 cm deep) filled with tap water maintained at the designated group temperature (5 ± 2 °C or 36 ± 2 °C). Rats were weighed before and after each week of the experiment. Resting rectal temperature was measured at baseline and after eight weeks. In the fourth week, the effect of a single swimming session on body temperature was also assessed in all groups.

Table 1 summarizes details of the swimming exercise protocol, including group size, session duration, and water temperature.

### 2.2. Procedures and Sample Preparation

At the end of the experiment, all rats were anesthetized with ketamine (10 mg/1000 g body weight) 48 h after the final training session. Soleus muscle samples were excised and immediately frozen in liquid nitrogen. The biological material was stored at −80 °C until further biochemical analyses were performed. Storage time did not exceed three months. Muscle tissue samples (100 mg) were manually ground in a mortar to obtain powdered tissue. This was followed by mechanical homogenization in 1× phosphate-buffered saline (PBS) and containing a cocktail of protease and phosphatase inhibitors (Cell Signaling Technology, Inc., Danvers, MA, USA, cat. 5872). The homogenates were then centrifuged (20 min; 4 °C; 2000 RCF). Total protein concentration in the homogenates was determined using the bicinchoninic acid (BCA) assay (Abcam, Cambridge, UK, cat. ab207002). The resulting supernatant was used to determine the concentrations of lipid peroxidation biomarkers (TBARS and 8-isoprostane), antioxidant enzymes (SOD1, CAT, GPx, GSSG-R, GST) and pPFK-1 (Ser775) expression.

### 2.3. Biochemical Analyses

#### 2.3.1. Measurement of 8-Isoprostane and TBARS Levels in Muscle

Muscle levels of 8-isoprostane and TBARS were determined using ELISA kits according to the manufacturer’s instructions: 8-isoprostane (Sunredbio, Shanghai, China), sensitivity: 0.608 ng/L; assay range: 0.7–180 ng/L. TBARS (Sunredbio, Shanghai, China), sensitivity: 0.486 nmol/mL; assay range: 0.5–100 nmol/mL

#### 2.3.2. Measurement of SOD1, CAT, GPx, GSSG-R, and GST Levels in Muscle

Muscle concentrations of selected antioxidant enzymes were measured using ELISA kits, following the manufacturers’ protocols: SOD1 (Sunredbio, Shanghai, China), sensitivity: 0.415 ng/mL; assay range: 0.5–100 ng/mL. CAT (FineTest, Wuhan, China), sensitivity: <18.75 mIU/mL; assay range: 31.25–2000 mIU/mL. GPx (Sunredbio, Shanghai, China), sensitivity: 0.723 ng/mL; assay range: 0.8–200 ng/mL. GST (Sunredbio, Shanghai, China), sensitivity: 0.715 ng/mL; assay range: 0.8–200 ng/mL. GSSG-R (Sunredbio, Shanghai, China), sensitivity: 0.475 ng/mL; assay range: 0.5–100 ng/mL.

#### 2.3.3. Analysis of pPFK-1 (Ser775) Expression by Western Blot

Protein separation was performed using SDS-PAGE (8–16% SurePAGE™, Bis-Tris gel, cat. M00660, GenScript USA Inc., NJ, USA), with 20 μg of protein loaded per well. Proteins were transferred to a 0.2 μm PVDF membrane (cat. 88520, Thermo Fisher Scientific, Waltham, MA, USA) using wet transfer (60 min, constant 70 V, Tris–glycine transfer buffer containing 15% methanol). Membranes were blocked in 5% skim milk for 60 min before antibody incubation in room temperature. Membranes were blocked in 5% skim milk for 60 min before antibody incubation.

Protein expression was assessed using an anti-PFK-1 (Ser775) antibody (cat. SL16457R, Sunlong Biotech, Hangzhou, China) at 1:500 dilution, and a secondary HRP-conjugated goat anti-rabbit IgG antibody (cat. ab97051, (Abcam, Cambridge, UK). GAPDH was used as a loading control, detected with anti-GAPDH antibody (cat. ab8245, Abcam, UK) and anti-mouse secondary antibody (cat. ab6789, Abcam, UK). Protein loading was performed based on BCA results and normalized using GAPDH rather than total protein staining; whilst exposure times were optimized to remain within the linear detection range, however, verification of signal linearity for this experiment was not performed.

Bands were visualized using WESTAR ANTARES ECL substrate (cat. XLS142, Cyanagen, Bologna, Italy) and imaged with a Molecular Imager ChemiDoc XRS+ system (Bio-Rad, Hercules, CA, USA). Six samples (*n* = 6) from each group were analyzed.

#### 2.3.4. Gene Expression Analysis of MAS Components by qRT-PCR

Quantitative assessment of mRNA expression for MAS enzymes was performed using real-time quantitative polymerase chain reaction (qRT-PCR). The target genes included cytosolic and mitochondrial NAD(H)-dependent malate dehydrogenases (*MDH1* and *MDH2*; EC 1.1.1.37), cytosolic and mitochondrial aspartate aminotransferases (*GOT1* and *GOT2*; EC 2.6.1.1), the mitochondrial alpha-ketoglutarate/malate carrier (*SLC25A11*), and two isoforms of the aspartate–glutamate carrier: *SLC25A12* (AGC1, also known as Aralar) and *SLC25A13* (AGC2, also known as Citrin).

Total RNA was extracted from muscle tissue stored at −80 °C using the RNeasy Mini Kit (Qiagen, Hilden, Germany). RNA quantity and purity were assessed with a NanoDrop ND-1000 spectrophotometer (NanoDrop Technologies, Wilmington, DE, USA). Reverse transcription was performed using 1 μg of RNA in a total volume of 20 μL with the Omniscript RT Kit (Qiagen, Germany), following the manufacturer’s instructions.

qRT-PCR was conducted using the 7500 Fast Real-Time PCR System (Applied Biosystems, Foster City, CA, USA) and Power SYBR Green PCR Master Mix (Applied Biosystems, USA), which contains AmpliTaq Gold DNA polymerase, a dNTP mixture, SYBR Green dye, and ROX as a passive reference. Fluorescence was measured in real time and was proportional to the PCR product concentration.

Each sample was analyzed in duplicate, and results were averaged. The reaction mixture contained 12.5 μL of Power SYBR Green Master Mix, forward and reverse primers at a final concentration of 0.5 μM, and 50 ng of cDNA, in a final volume of 25 μL. The thermal cycling protocol included an initial step at 95 °C for 15 s (activation), followed by 40 cycles of 95 °C for 15 s (denaturation) and 60 °C for 1 min (annealing/extension).

Gene expression levels were normalized to an endogenous control. Reaction specificity was confirmed by melt curve analysis, which verified the amplification of a single PCR product. Primer sequences for all target genes were as follows:
name: rMDH1_Fsequence: GAGCCAATCAGAGTCCTCGTGACname: rMDH1_Rsequence: GGCACAGTCTTGCAGTTCCAname: rMDH2_Fsequence: GCAACCCCTTTCACTCCTCname: rMDH2_Rsequence: TCTGGTCTCGATGTGACTCAGATname: rGOT1_Fsequence: GAGCGTACCGCACAGATGACTname: rGOT1_Rsequence: GACTGTGGTCGTTAGCAATCTTname: r SLC25A11_Fsequence: GTACCTCCCCTAAGTCTGTCAAname: r SLC25A11_Rsequence: CTGCATCCGGTTCTTCACCAGname: r SLC25A12_Fsequence: ATGGCGGTCAAGGTGCACAC.name: r SLC25A12_Rsequence: AGCGTTGAACGAAGTCTTCCGname: r SLC25A13_Fsequence: CAGCCCAACCCGAAGACTGTname: r SLC25A13_Rsequence: CTGGAAGGCCACCATAAACAA

The reference gene used for normalization was *GAPDH*. Absolute gene expression was calculated using the 2^–ΔCt method, where ΔCt = (Ct of the target gene) − (Ct of the reference gene); Ct denotes the threshold cycle.

#### 2.3.5. Statistical Analysis

Statistical analysis was performed using STATISTICA software (v.12.5 PL). In addition to descriptive statistics (mean, standard deviation, median, upper and lower quartiles, minimum and maximum), the normality of distribution of the analyzed parameters was assessed using the Shapiro–Wilk test. As variables not following a normal distribution, the Kruskal–Wallis one-way ANOVA and next the Mann–Whitney U test were used to compare experimental groups. A significance level of *p* ≤ 0.05 was considered statistically significant. Data are presented as mean and standard deviation. In order to investigate the effect of sex on the tested parameters statistical analyses were conducted separately for each of the dependent variables. For each endpoint, a two-way robust analysis of variance (robust ANOVA) was applied using a Sex (male vs. female) × Group (36 °C, 5 °C, control) factorial design. The procedure employed bootstrap resampling (599 replications), a modified one-step estimator, and projection distances to reduce the influence of outliers and to mitigate violations of the assumptions underlying classical ANOVA. Because adjusted critical values were used, the omnibus-effects tables report *p*-values only. Following the omnibus test, post hoc comparisons were performed for: (i) the main effect of Sex; (ii) the main effect of Group (pairwise contrasts); (iii) simple effects within the Sex × Group interaction. Results were reported as ψ-hat (a robust estimate of location difference), 95% confidence intervals (bootstrap-based), and corresponding *p*-values. All analyses were performed in jamovi v2.6 using R v4.4.

## 3. Results

### 3.1. Concentrations of Lipid Peroxidation Biomarkers in Rat Skeletal Muscle

#### 3.1.1. TBARS Concentration

**Figure 2:**
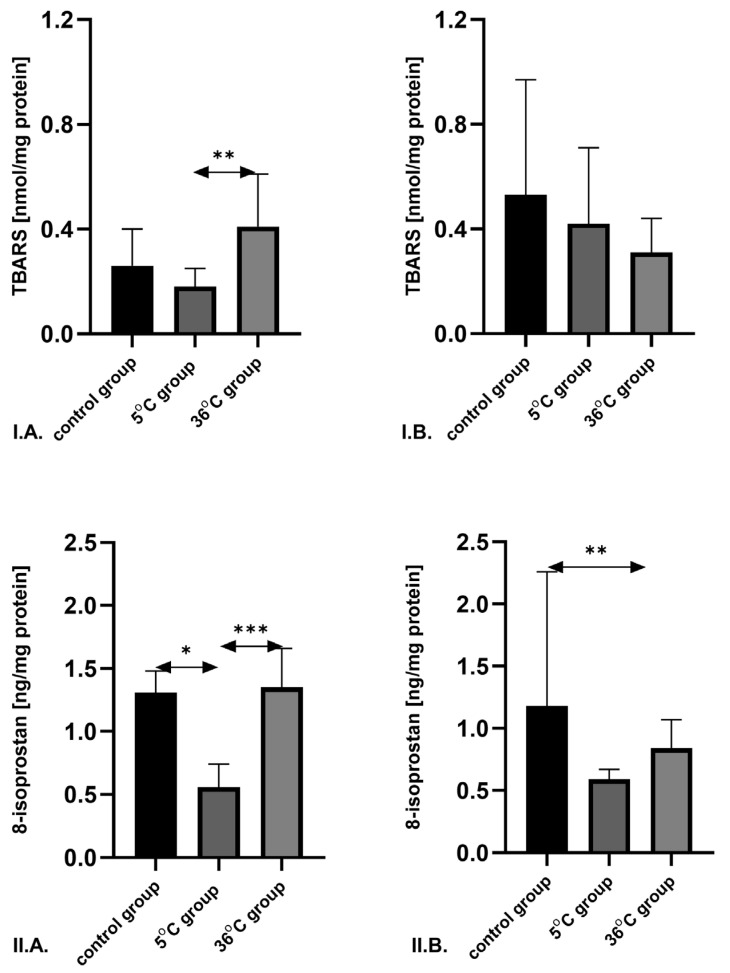
TBARS (**I**) and 8-isoprostane (**II**) concentrations in the skeletal muscles of male (**A**) and female (**B**) rats from the control and experimental groups. Rats underwent swimming training in cold water (5 °C) or thermoneutral water (36 °C) for nine weeks. Sedentary rats served as controls. In the first week, swimming began at 2 min on day one and increased by 0.5 min daily, reaching 4 min by day five. Data are presented as means ± standard deviation. * *p* ≤ 0.05 denotes significance compared to the control group (Mann–Whitney U test); ** *p* ≤ 0.01 denotes significance compared to the control group (Mann–Whitney U test); *** *p* ≤ 0.001 denotes significance compared to the control group (Mann–Whitney U test). The robust two-way ANOVA provided no evidence of a Sex × Group interaction (all interaction *p*-values were ≥0.05).

The concentration of TBARS in the skeletal muscles of male rats subjected to cold-water swimming was significantly lower—by approximately 45.9% (*p* = 0.027)—compared to that in males trained in thermoneutral water. In female rats, no significant differences in TBARS levels were observed between the groups. The robust two-way ANOVA provided no evidence of a Sex × Group interaction (all interaction *p*-values were ≥0.05) (Figure 2).

#### 3.1.2. 8-Isoprostane Concentration

In male rats, the 8-isoprostane (8-IsoP) concentration was significantly reduced in those trained in cold water compared to both the thermoneutral group (by 56.0%; *p* = 0.001) and the sedentary control group (by 52.3%; *p* = 0.013).

In female rats, 8-IsoP levels in the muscles of those swimming in thermoneutral water were significantly lower than in the control group (by 14.9%; *p* = 0.048). The robust two-way ANOVA provided no evidence of a Sex × Group interaction (all interaction *p*-values were ≥0.05) (Figure 2).

### 3.2. Antioxidant Enzyme Concentration in the Skeletal Muscles of Male and Female Rats

**Figure 3:**
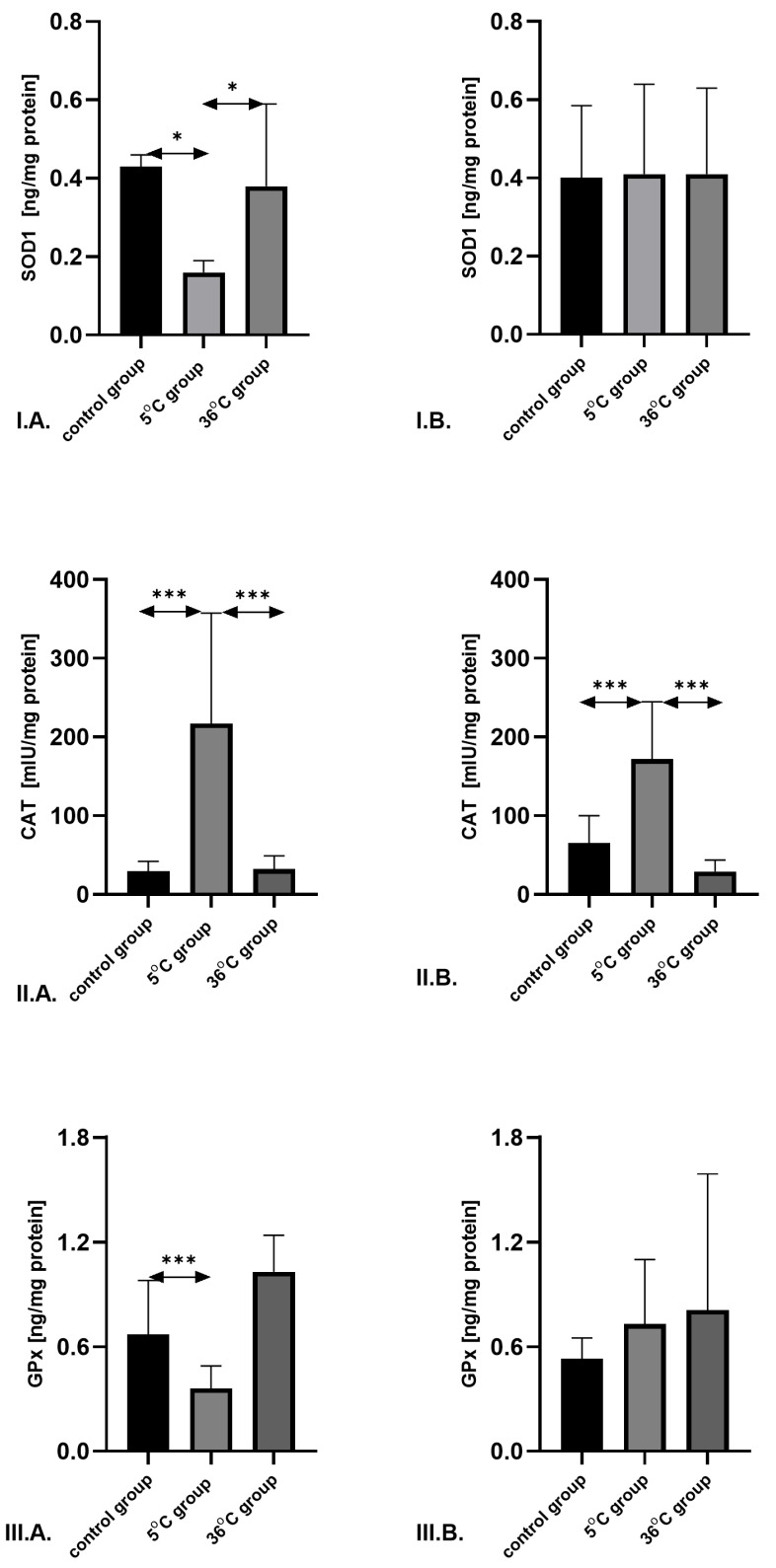
Concentrations of SOD1 (**I**), CAT (**II**), and GPx (**III**) in the skeletal muscles of male rats (**A**) and female rats (**B**) from the control and experimental groups. Rats underwent swimming training in cold water (5 °C) or thermoneutral water (36 °C) for nine weeks. The control group remained sedentary. During the first week of the protocol, the initial swimming session lasted 2 min on day one and was extended by 0.5 min per day, reaching 4 min by day five. Data are presented as means ± standard deviation. * *p* ≤ 0.05 denotes significance compared to the control group (Mann–Whitney U test); *** *p* ≤ 0.001 denotes significance compared to the control group (Mann–Whitney U test). The robust two-way ANOVA provided no evidence of a Sex × Group interaction (all interaction *p*-values were ≥0.05).

SOD1 concentration in males was significantly lower in the cold-water group compared to both the thermoneutral group (by 56.4%; *p* = 0.02) and the control group (by 59.5%; *p* = 0.04). No significant differences in SOD1 concentration were observed among the female groups (Figure 3).

CAT concentration in males was significantly higher in the cold-water group compared to the control group (by 640%; *p* = 0.001) and the thermoneutral group (by 570%; *p* = 0.0001). In females, cold-water swimming induced a pronounced increase in CAT concentration compared to the control (by 162%; *p* = 0.0001) and thermoneutral groups (by 480%; *p* = 0.0001) (Figure 3).

GPx concentration was significantly lower in males swimming in cold water compared to the control group (by 65.1%; *p* = 0.0003). No significant differences in GPx concentration were observed among female groups (Figure 3).

**Figure 4:**
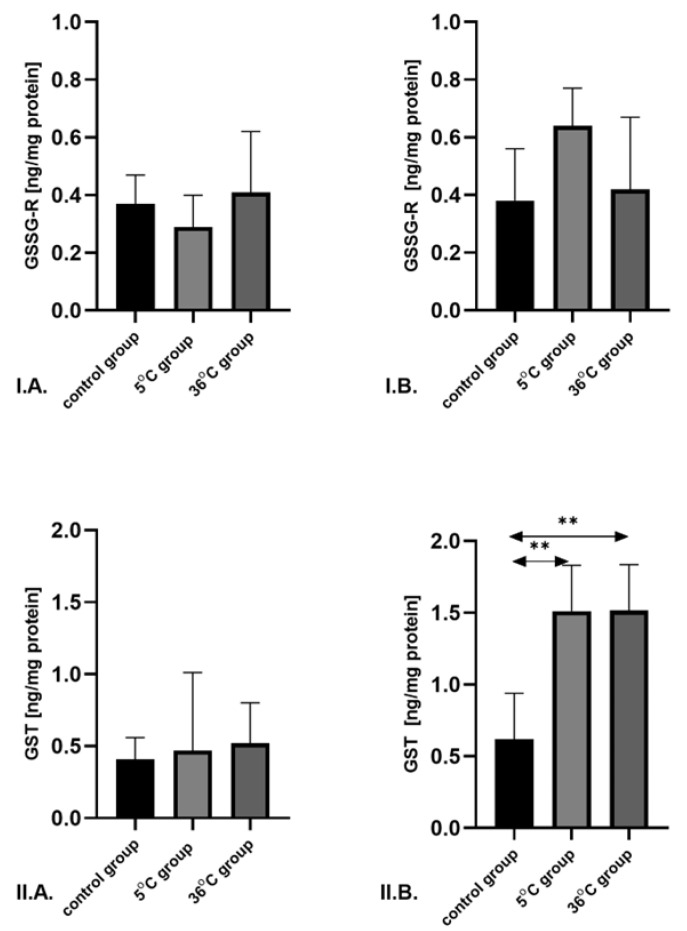
Concentrations of GSSG-R (**I**) and GST (**II**) in the skeletal muscles of male rats (**A**) and female rats (**B**) from the control and experimental groups. Rats underwent swimming training in cold water (5 °C) or thermoneutral water (36 °C) for nine weeks. The control group remained sedentary. During the first week of the study, the initial swimming session lasted 2 min on day one and was extended by 0.5 min per day, reaching 4 min by day five. Data are presented as means ± standard deviation. ** *p* ≤ 0.01 denotes significance compared to the control group (Mann–Whitney U test). The robust two-way ANOVA provided no evidence of a Sex × Group interaction (all interaction *p*-values were ≥0.05).

GSSG-R concentration showed no differences among male and both female groups. The robust two-way ANOVA provided no evidence of a Sex × Group interaction (all interaction *p*-values were ≥0.05) (Figure 4).

GST concentration was significantly lower in females in the cold-water group compared to controls (by 42%; *p* = 0.001), and significantly lower in the thermoneutral group compared to controls (by 59%; *p* = 0.001). No differences in GST expression were observed among male groups (Figure 4).

### 3.3. Malate–Aspartate Shuttle (MAS) mRNA Expression

#### 3.3.1. Expression of MDH1 and MDH2 in Rat Muscles

**Figure 5:**
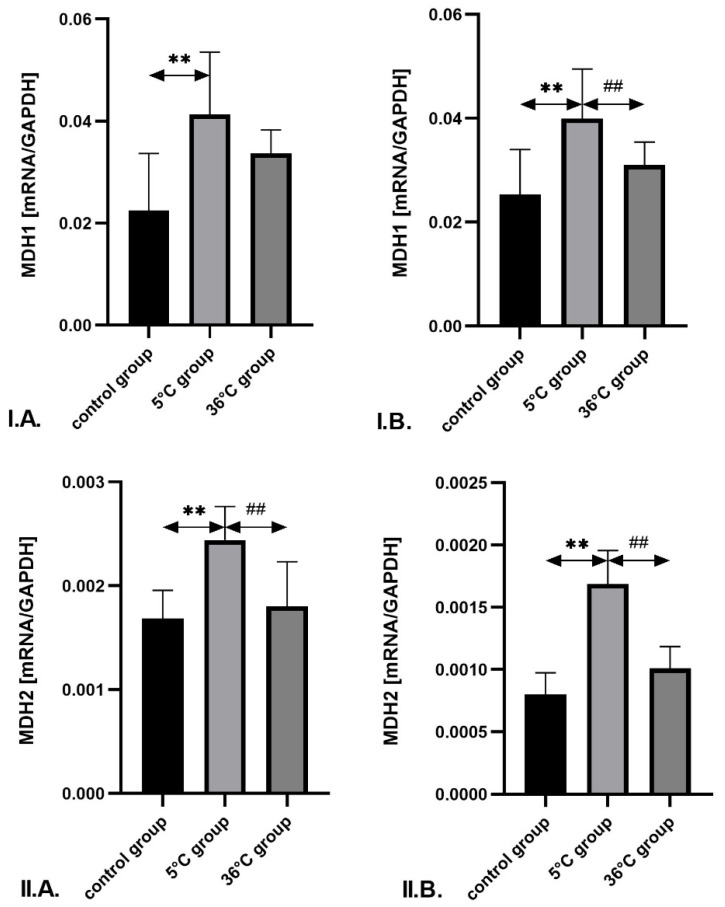
Expression of ***MDH1*** (**I**) and ***MDH2*** mRNA (**II**) in the skeletal muscles of male rats (**A**) and female rats (**B**) from the control and experimental groups. Rats underwent swimming training in cold water (5 °C) or thermoneutral water (36 °C) for nine weeks. The control group remained sedentary. During the first week of the study, the initial swimming session lasted 2 min on day one and was extended by 0.5 min per day, reaching 4 min by day five. Data are presented as means ± standard deviation. ** *p* ≤ 0.01 denotes significance compared to the control group (Mann–Whitney U test); ## *p* ≤ 0.01 indicates a significant difference compared to the 5 °C group (Mann–Whitney U test). The robust two-way ANOVA provided no evidence of a Sex × Group interaction (all interaction *p*-values were ≥0.05).

The expression of *MDH1* and *MDH2* mRNA in the skeletal muscles of male rats swimming in cold water was significantly higher—by approximately 83% (*p* = 0.001) and 26% (*p* = 0.001), respectively—compared to the control group. A similar increase in *MDH2* mRNA expression was observed in cold-water-trained males compared to those trained in thermoneutral water (by approximately 18%; *p* = 0.002). In female rats undergoing cold-water training, the expression of *MDH1* and *MDH2* mRNA was also significantly higher compared to the control group—by approximately 55% (*p* = 0.001) and 110%, respectively. Furthermore, cold-water training resulted in significantly higher expression of *MDH1* and *MDH2* mRNA in female rats compared to thermoneutral training (by approximately 59%; *p* = 0.002 and 66%; *p* = 0.001, respectively). No significant differences were observed between the thermoneutral group and control group in females (Figure 5).

#### 3.3.2. Expression of Got1 and Got2 mRNA in Rat Muscles

**Figure 6:**
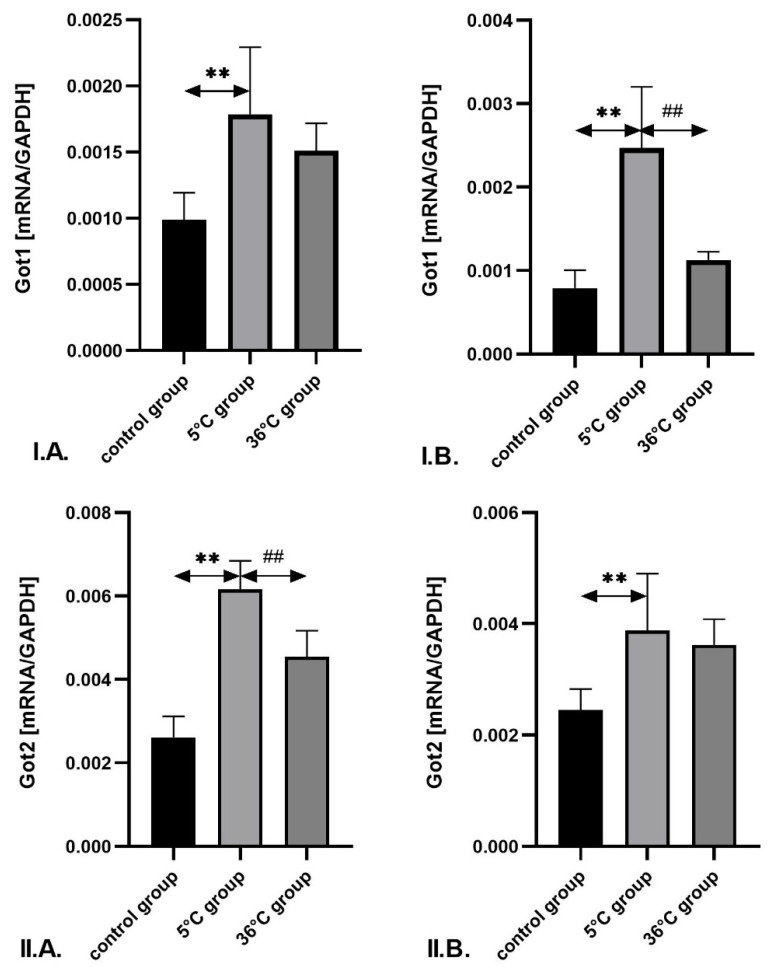
Expression of *Got1* (**I**) and *Got2* mRNA (**II**) in the skeletal muscles of male rats (**A**) and female rats (**B**) from the control and experimental groups. Rats underwent swimming training in cold water (5 °C) or thermoneutral water (36 °C) for nine weeks. The control group remained sedentary. During the first week of the study, the initial swimming session lasted 2 min on day one and was extended by 0.5 min per day, reaching 4 min by day five. Data are presented as means ± standard deviation. ** *p* ≤ 0.01 denotes significance compared to the control group (Mann–Whitney U test); ## *p* ≤ 0.01 indicates a significant difference compared to the 5 °C group (Mann–Whitney U test). The robust two-way ANOVA provided no evidence of a Sex × Group interaction (all interaction *p*-values were ≥0.05).

In male rats, the expression of *Got1* mRNA in the skeletal muscle was significantly higher in the cold-water group compared to the control group (by approximately 81%; *p* = 0.001). Additionally, *Got2* mRNA expression in cold-water-trained males was significantly higher compared to both the control group (by approximately 136%; *p* = 0.002) and the thermoneutral group (by 36%; *p* = 0.001). In females undergoing cold-water training, *Got1* and *Got2* mRNA expression in the muscle was significantly higher than in controls—by approximately 213% (*p* = 0.002) and 37% (*p* = 0.002), respectively. Furthermore, *Got1* mRNA expression in cold-water-trained females was significantly higher (by approximately 119%; *p* = 0.001) compared to those trained in thermoneutral water (Figure 6).

#### 3.3.3. Expression of *Ogc*/*SLC25A11* mRNA in Rat Muscles

In the skeletal muscles of male rats trained in cold water, the mRNA expression of *Ogc*/*SLC25A11* was significantly higher—by approximately 72% (*p* = 0.001)—compared to the control group.

**Figure 7:**
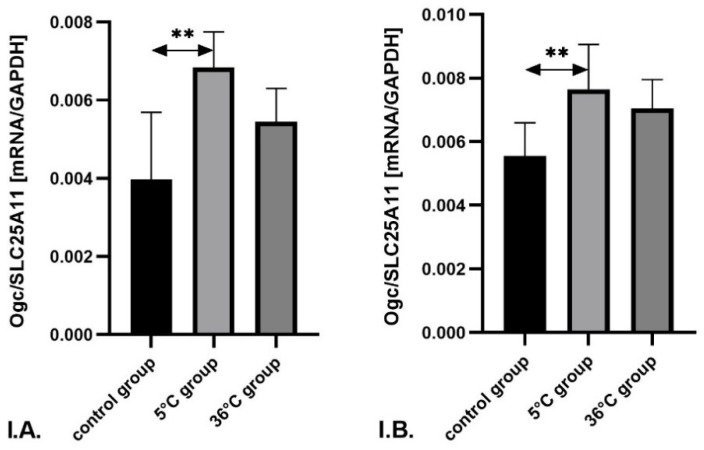
Expression of *Ogc*/*SLC25A11* (**I**) mRNA in the skeletal muscles of male rats (**A**) and female rats (**B**) from the control and experimental groups. Rats underwent swimming training in cold water (5 °C) or thermoneutral water (36 °C) for nine weeks. The control group remained sedentary. During the first week of the study, the initial swimming session lasted 2 min on day one and was extended by 0.5 min per day, reaching 4 min by day five. Data are presented as means ± standard deviation. ** *p* ≤ 0.01 denotes significance compared to the control group (Mann–Whitney U test). The robust two-way ANOVA provided no evidence of a Sex × Group interaction (all interaction *p*-values were ≥0.05).

In female rats undergoing cold-water training, *Ogc*/*SLC25A11* mRNA expression was also significantly higher—by approximately 38% (*p* = 0.002)—compared to the control group (Figure 7).

#### 3.3.4. Expression of AGC1 and AGC2 mRNA in Rat Muscles

The mRNA expression of *AGC1* and *AGC2* in the skeletal muscles of male rats trained in cold water was significantly higher—by approximately 145% (*p* = 0.001) and 140% (*p* = 0.002), respectively—compared to the control group.

**Figure 8:**
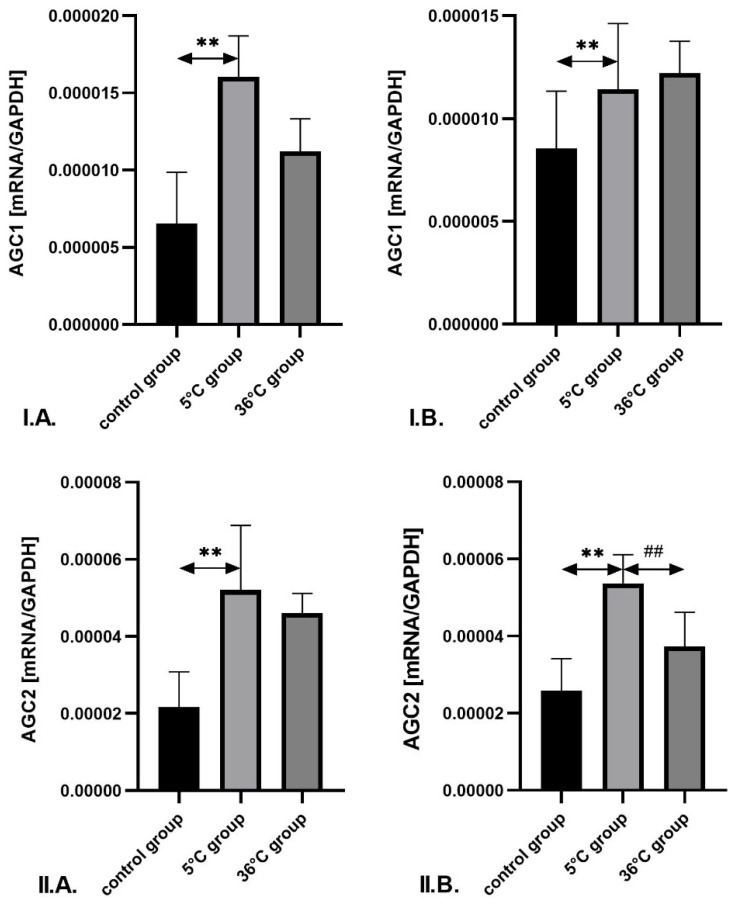
Expression of *AGC1* (**I**) and *AGC2* mRNA (**II**) in the skeletal muscles of male rats (**A**) and female rats (**B**) from the control and experimental groups. Rats underwent swimming training in cold water (5 °C) or thermoneutral water (36 °C) for nine weeks. The control group remained sedentary. During the first week of the study, the initial swimming session lasted 2 min on day one and was extended by 0.5 min per day, reaching 4 min by day five. Data are presented as means ± standard deviation. ** *p* ≤ 0.01 denotes significance compared to the control group (Mann–Whitney U test); **##** *p* ≤ 0.01 indicates a significant difference compared to the 5 °C group (Mann–Whitney U test). The robust two-way ANOVA provided no evidence of a Sex × Group interaction (all interaction *p*-values were ≥0.05).

In female rats undergoing cold-water training, the expression of *AGC1* and *AGC2* mRNA was also significantly higher—by approximately 34% (*p* = 0.002) and 108% (*p* = 0.002), respectively—compared to the control group. Furthermore, in the muscles of cold-water-trained females, *AGC2* mRNA expression was significantly higher (by approximately 44%; *p* = 0.001) than in the thermoneutral group (Figure 8).

#### 3.3.5. Expression of Phosphofructokinase-1 in Rat Muscles

**Figure 9:**
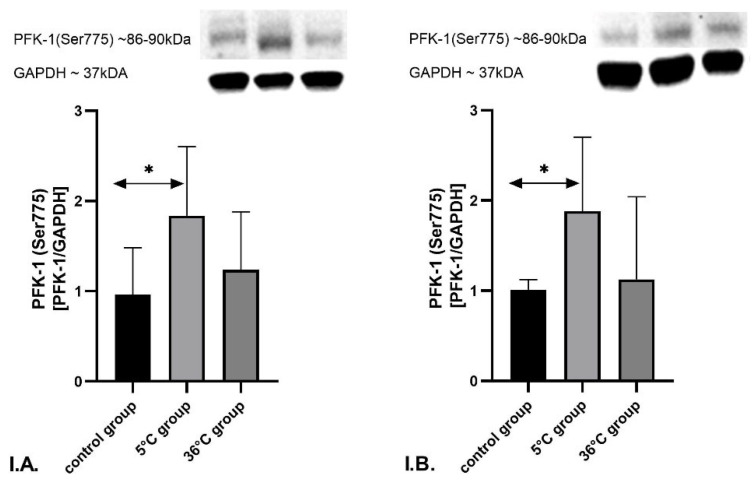
Representative Western blots and densitometric analysis of PFK-1 (Ser775) (**I**) protein expression levels (normalized to GAPDH) in the skeletal muscles of male rats (**A**) and female rats (**B**) from the control and experimental groups. Rats underwent swimming training in cold water (5 °C) or thermoneutral water (36 °C) for nine weeks. The control group remained sedentary. During the first week of the study, the initial swimming session lasted 2 min on day one and was extended by 0.5 min per day, reaching 4 min by day five. Data are presented as means ± standard deviation. * *p* ≤ 0.05 denotes significance compared to the control group (Mann–Whitney U test). The robust two-way ANOVA provided no evidence of a Sex × Group interaction (all interaction *p*-values were ≥0.05).

A statistically significant increase in PFK-1 (Ser775) expression was observed in the skeletal muscles of male rats in the 5 °C group compared to the control group (*p* = 0.04). The 36 °C group exhibited a moderate but not statistically significant elevation in the protein expression relative to control. No significant differences were found between the 36 °C and 5 °C groups (Figure 9(I.A.)).

Similar results were observed in the skeletal muscles of female rats. A densitometric quantification demonstrated a significant increase in the expression of PFK-1 (Ser775) in the 5 °C group versus the control group (*p* = 0.04). There were no significant differences in the PFK-1 (Ser775) expression between the 36 °C group and control group, and between the 36 °C group and the 5 °C group (Figure 9(I.B.))

## 4. Discussion

In this study, we aimed to determine whether cold-water swimming could serve as a potential strategy to enhance antioxidant capacity, improve NADH utilization in oxidative metabolism, and consequently lead to better muscle metabolism, improved mitochondrial function, and increased ATP production in the skeletal muscles, as observed in our previous study [7].

Our findings revealed increased mRNA expression of all MAS enzymes involved in the delivery of NADH to mitochondria and elevated expression of the active form of phosphofructokinase 1—indicating intensified aerobic glycolysis, increased ROS production, and upregulation of antioxidant enzymes. These all suggest improved mitochondrial performance in the muscles of older adult rats subjected to cold-water swimming training.

Although the observed changes in mRNA levels of MAS components may reflect adaptive responses over the 6-week training period, they cannot be directly assumed to correspond to proportional protein expression or increased MAS flux without proteomic validation.

### 4.1. Adaptation to Cold-Water Swimming and Antioxidant Balance

Exposure to ambient temperatures below thermal comfort thresholds induces thermal stress in the organism, but when repeated, it may trigger adaptive responses consistent with the concept of hormesis [12]. Low temperatures combined with physical exertion lead to increased heat production, accompanied by elevated cellular respiration, oxygen consumption, and ROS generation, along with altered antioxidant enzyme activity [15]. It is also known that cold exposure enhances the activity of enzymes involved in aerobic metabolism and increases tissue oxygen consumption in animal models [25,26,27]. Chronic cold exposure raises metabolic rate in mammals, leading to hypertrophy of metabolically active tissues such as brown and white adipose tissue, liver, kidneys, small intestine, and heart, with adaptive changes varying by sex [13,14,15,19]. ROS production and oxidative stress may contribute to oxidative damage that plays a significant role in the aging process [8]. At appropriate levels, ROS are important intracellular signaling molecules. Cells exposed to ROS or xenobiotics that generate ROS activate multiple signalling pathways, including those involved in prostanoid synthesis and inflammation development in animal models [28,29,30,31,32]. However, excessive ROS levels lead to lipid peroxidation [33,34].

In our study, the concentrations of lipid peroxidation markers—TBARS and 8-isoprostane—were lowest in the muscles of male rats trained in cold water and significantly lower than those in males trained in warm water. TBARS levels in cold-water-trained males were also significantly lower than in females. No significant differences in TBARS levels were observed among the female groups; however, the lowest 8-isoprostane concentration was found in the muscles of cold-water-trained females. In addition, 8-isoprostane levels in females trained in warm water were significantly lower than in males undergoing the same training condition. However, in our study, the robust two-way ANOVA provided no evidence of a Sex × Group interaction (all interaction *p*-values were ≥0.05) across all studied parameters. Within the limits of this dataset and the robust bootstrap approach, sex did not moderate the effect of training condition (36 °C vs. 5 °C vs. control) on these transcripts—i.e., the pattern of group differences was broadly comparable in males and females—and any observed sex differences are better framed as main effects rather than differential training responses by sex. However, studies by other authors have shown that female rats produce half as much superoxide as males [35]. The expression and activity of mitochondrial antioxidant enzymes in females were higher than in males of the same chronological age [35]. Female mitochondria produce significantly less hydrogen peroxide than male mitochondria and have higher levels of mitochondrial reduced glutathione, manganese superoxide dismutase, and GPx than male mitochondria [35]. Oxidative damage to mitochondrial DNA is also four times higher in men than in women. These differences may be due to estrogen action, but this requires further study, particularly in the context of cold-water exercise [36]. Dede et al. [37] reported that acute hypothermia caused by short-term cold-water immersion resulted in increased levels of another oxidative stress marker—malondialdehyde (MDA)—in the muscles of male rats. However, it has been shown that repeated exposure to low levels of oxidative stress may induce adaptive changes that help organisms resist stress-induced damage. Antioxidant protection has been demonstrated to improve as an adaptive response to repeated cold-induced oxidative stress in humans [38].

### 4.2. The Impact of ROS on Tissues Is Closely Related to the Antioxidant Defense Capacity Mediated by Enzymes Such as SOD1, CAT, GPx, GSSG-R, and GST

SOD1 catalyzes the dismutation of superoxide into hydrogen peroxide, which is subsequently converted to H_2_O by CAT and GPx [39]. An imbalance in antioxidant enzyme activity may play a key role in the pathology of ROS-related damage. This is particularly relevant for the balance between SOD1 and CAT activities, often assessed as the SOD1/CAT ratio. Excessive SOD1 activity relative to CAT, in the presence of Fe^2+^ and Cu^2+^, may promote the formation of highly reactive hydroxyl radicals. Conversely, insufficient SOD1 activity may also lead to hydroxyl radical production via the Haber–Weiss reaction [40]. A disturbed balance between the activities of these enzymes is unfavorable, particularly under conditions of elevated oxidative stress and excessive ROS synthesis, including hydrogen peroxide, and may increase the extent and severity of tissue damage [41].

Changes in the balance between antioxidant enzyme activities, expressed as SOD1/CAT and SOD1/GPx ratios, may indicate reduced efficacy of tissue protection against excessive hydrogen peroxide levels. In our study, we observed high expression of CAT and a low SOD1/CAT ratio in the muscles of rats trained in cold water, which may reflect effective antioxidant protection and support the notion of its enhancement as an adaptive response to repeated cold-induced oxidative stress. The balanced SOD1/GPx expression observed in the muscles of aging female rats swimming in cold water further supports this interpretation. GPx has a higher affinity for hydrogen peroxide and therefore plays a more prominent role when hydrogen peroxide concentrations are relatively low; reduced activity of GPx or CAT can be mutually compensated [41].

### 4.3. Malate–Aspartate Shuttle (MAS) mRNA Expression and Improvement of Mitochondrial Metabolism

In our study, we demonstrated that cold-water swimming increases the mRNA expression of all components of the malate–aspartate shuttle in male and female rats, which contributes to more efficient NADH utilization in oxidative metabolism, improved muscle metabolism, and enhanced mitochondrial function. We also observed increased expression of the active (phosphorylated) form of phosphofructokinase-1, a key regulatory enzyme in glycolysis, which under aerobic conditions is an important source of NADH for the MAS.

To our knowledge, no previous studies have reported on the effect of cold-water immersion on the expression of MAS components, and thus our findings provide novel evidence on the NADH transport system, supporting the hypothesis of beneficial physiological effects of cold-water immersion. In contrast, studies investigating MAS expression in response to resistance training in trained and untrained individuals using muscle biopsies of the deltoid muscle found no significant changes in the expression of MAS enzymes, nor in Krebs cycle or glycolytic enzymes [42]. These results support our hypothesis that cold-water training leads to increased glycolytic intensity (as indicated by elevated phosphorylated phosphofructokinase-1 expression) and enhanced mitochondrial NADH utilization (reflected by upregulation of all MAS components).

In a subsequent study by Schantz et al. [43,44], which aimed to assess whether endurance training affects MAS enzyme levels in human skeletal muscle, muscle biopsies from the lateral portion of the vastus lateralis were analyzed in both sedentary individuals and endurance-trained subjects. Results showed that MAS enzyme levels were approximately 50% higher in the trained state—with cytoplasmic malate dehydrogenase elevated by 36%, mitochondrial malate dehydrogenase by 46%, cytoplasmic aspartate aminotransferase by 52%, and mitochondrial aspartate aminotransferase by 48%. The authors concluded that endurance training increases MAS enzyme levels.

In another study investigating MAS enzyme expression in type I (slow-twitch) and type II (fast-twitch) fibers of the vastus lateralis muscle, and the effect of endurance training in both untrained and endurance-trained individuals, it was shown that MAS enzymes are more highly expressed in type I than in type II fibers. The same researchers also demonstrated that, unlike MAS enzymes, the α-glycerophosphate shuttle is more prominently expressed in type II fibers and appears unaffected by endurance training [42]. These findings further support our hypothesis that cold-water training positively influences mitochondrial NADH utilization.

The effect of cold on MAS enzymes is also indicated by recent findings in brown adipose tissue (BAT) [45]. The authors reported that the malate–aspartate shuttle is activated by cold in BAT and plays a crucial role in promoting mitochondrial fatty acid utilization. The mechanism proposed involves cold-induced upregulation of glutamate–oxaloacetate transaminase (GOT1), a key MAS enzyme, through the β-adrenergic receptor–PKA–PGC-1α signaling axis. Increased GOT1 activity enhances MAS function by transporting reduced equivalents from the cytosol to mitochondria. This process augments mitochondrial fatty acid oxidation while limiting glucose oxidation under cold conditions. In our study, we also observed increased *GOT1* expression during cold-water training, accompanied by elevated phosphofructokinase-1 expression, indicating intensified glycolysis.

Improvement in mitochondrial energy metabolism in the muscles of older adult rats as a result of cold-water swimming was also confirmed by findings from our previous study conducted using the same experimental model [7]. We showed that swimming in thermoneutral water improved the energy metabolism of skeletal muscles, as evidenced by elevated ATP, ADP, TAN (total adenine nucleotide pool), and AEC (adenylate energy charge) concentrations, along with increased mRNA expression of proteins regulating mitochondrial biogenesis (such as PGC-1α) and fusion. Similarly, cold-water swimming enhanced muscle energy metabolism in rats, reflected in increased energy turnover and upregulation of mitochondrial biogenesis and dynamics [7].

It appears that MAS transporter expression, along with concentrations of high-energy compounds and the expression of proteins regulating mitochondrial dynamics in muscle tissue, may serve as valuable indicators for monitoring adaptive changes in muscles during cold-water exercise. However, due to the high popularity of this form of active leisure, combined with the lack of medical supervision and a limited number of studies with confirmed physiological effects, there is a clear need for further research to explore and validate hypotheses in this area.

Moreover, the observed elevations in mRNA expression of MAS components likely reflect adaptive responses to six weeks of cold-water swimming, potentially mediated by transcriptional regulators and increased metabolic demand. Nevertheless, changes at the mRNA level do not necessarily translate into proportional alterations in protein abundance or activity, owing to post-transcriptional regulation, differences in mRNA stability, and variable protein turnover rates. Such mechanisms may uncouple transcriptomic and proteomic responses in this model. For example, acute cold exposure can induce transient increases in mRNA expression that are not accompanied by sustained changes at the protein level. At the same time, rapid transcriptional responses are considered an important early step in muscle adaptation that may precede functional remodelling at the protein level [46,47]. Taken together, our findings suggest a potential upregulation of the malate–aspartate shuttle that may extend beyond the transcriptomic level; however, direct assessment of protein expression is required to confirm the functional consequences of cold-water training on mitochondrial NADH shuttling.

## 5. Conclusions

In this study, we observed that cold-water swimming was associated with increased mRNA expression of all analyzed components of the malate–aspartate shuttle in older male and female rats. These transcriptomic changes may indicate enhanced capacity for NADH transfer and oxidative metabolism in skeletal muscles. However, because the present analyses were limited to mRNA expression, further studies incorporating protein quantification and functional assays are necessary to determine whether these molecular alterations translate into improved mitochondrial function and muscle metabolic performance.

## Figures and Tables

**Table 1:** Summary of data from the experimental study.

Sex	N	Age [Months]	Duration of Experiment	Water Temperature	Duration of Swimming Exercise	
♂	12	15	8 weeks (5 day/week)	5 ± 2 °C	1st week [1–4 min]	2nd–8th week [4 min]
	12			36 ± 2 °C		
	8			Non-swimming control group		
♀	12			5 ± 2 °C	1st week [1–4 min]	2nd–8th week [4 min]
	12			36 ± 2 °C		
	8			Non-swimming control group		

## Data Availability

The original contributions presented in this study are included in the article. Further inquiries can be directed to the corresponding author.
